# Health-Seeking Behavior and Its Associated Technology Use: Interview Study Among Community-Dwelling Older Adults

**DOI:** 10.2196/43709

**Published:** 2023-05-04

**Authors:** Yichi Zhang, Edmund W J Lee, Wei-Peng Teo

**Affiliations:** 1 Physical Education and Sports Science Academic Group National Institute of Education Nanyang Technological University Singapore Singapore; 2 Ageing Research Institute for Society and Education Interdisciplinary Graduate Programme Nanyang Technological University Singapore Singapore; 3 Wee Kim Wee School of Communication and Information Nanyang Technological University Singapore Singapore

**Keywords:** health, health-seeking behavior, aging, technology, telehealth, mobile health, mHealth, eHealth, health access, qualitative study, mobile phone

## Abstract

**Background:**

Understanding older people’s health-seeking behavior (HSB) is crucial for uncovering their health needs and priorities and developing appropriate policies to address them and avert their disease progression. Technologies play an active role in our daily lives and have been incorporated into health activities to support the older population and facilitate their HSB. However, previous studies of HSB have mainly focused on behaviors during illness, and there are limited studies on how technologies have been used in older people’s health-seeking activities.

**Objective:**

This study aimed to investigate HSB and the associated technology use among the older population, ultimately proposing implications for practice to address their unmet health needs.

**Methods:**

This paper presents partial data from a large qualitative study, which has been approved by the institutional review board and used a phenomenological approach. Semistructured interviews were conducted between April 2022 and July 2022, either via Zoom (Zoom Video Communications Inc) or face-to-face sessions. Inclusion criteria were being aged ≥50 years, long-term residence in Singapore, and being able to speak English or Mandarin. The interviews were manually transcribed verbatim, and thematic analysis was performed, with the individual as the unit of analysis to understand the patterns of behaviors.

**Results:**

In total, 15 interviews were conducted to reach thematic saturation. We identified 5 main consequences of HSB, which were aligned with the original HSB model. Regarding technology use in health seeking, 4 themes were extracted: the most widely used digital technologies are the mobile health apps and wearable devices with the associated wellness programs launched by the government and local companies, and they have the potential to enhance health communication, promote health maintenance, and increase access to health services; information communication technologies and social media, though not primarily designed for health purposes, play a substantial role in easing the process of seeking health information and managing symptoms. Although the outbreak of the COVID-19 pandemic has resulted in some alterations to older adults’ well-being, it has catalyzed the adoption of telehealth as a complement to access health care services, and older adults have different considerations when selecting technologies to facilitate their health seeking and fulfill their health needs. Moreover, 4 archetypes were proposed based on our findings and the insights gained from our participants’ observations in their social networks. These findings led to several implications for practice regarding health communication and promotion, health education, technology design and improvement, telemonitoring service implementation, and solutions to address the needs of each proposed archetype.

**Conclusions:**

Unlike the commonly held belief that older adults resist technologies and lack technological proficiency, our findings showed that technologies could play a promising role in facilitating older adults’ health seeking. Our findings have implications for the design and implementation of health services and policies.

## Introduction

### Background

Health-seeking behavior (HSB) is an important concept associated with a country’s health status and economic development [1]. It also helps uncover the health needs and priorities of a population and informs the development of appropriate policies to address their needs and avert their disease progress [2]. In the literature, HSB has been studied with different scopes. The most commonly used definition is “any action or inaction undertaken by individuals who perceive themselves to have a health problem or to be ill for the purpose of finding an appropriate remedy” [3], and studies adopting this definition always examined the formal health system use or the process of illness responses [2,4-6]. However, one could argue that this definition focuses mainly on illness behaviors [7] and ignores the importance of promoting overall health and well-being, as health is *not merely the absence of disease or infirmity* but rather *a state of complete physical, mental, and social well-being* [8]. Thus, HSB could be viewed in a broader sense. A more comprehensive definition proposed by Chinn and Kramer is *an individual’s deed to the promotion of maximum well-being, recovery, and rehabilitation; this could happen with or without health concerns and within a range of potential to real health concerns* [9]. This definition encompasses practices for preserving optimal wellness, preventing illnesses, and addressing any deviation from good health [1,10], which aligns with the concept of universal health coverage [11].

Technologies have been seamlessly integrated into different aspects of people’s life and reshaped people’s health-seeking activities. For instance, smartwatches and wristbands with various sensors can collect continuous biological, behavioral, and environmental data; deliver health interventions; and measure users’ health outcomes [12]. Mobile apps can keep people connected with their families and friends, disseminate health information [13], store and share health and lifestyle data [14], manage chronic diseases [15], and manage medical appointments [16]. Self-test kits, such as Antigen Rapid Test Kits, allow people to obtain results swiftly and conveniently at any location and time [17]. As another example, telemedicine services such as telephone calls and video consultations have also demonstrated their potential to be a cost-effective and efficient alternative solution to access quality health care during the COVID-19 pandemic, by reducing travel time and protecting users from disease transmission [18].

Singapore has one of the fastest-growing older populations in the world owing to the increasing life expectancy and low fertility rates [19,20]. Statistics showed that the proportion of the silver generation in Singapore has been rising from 3.4% in 1970 to 10.4% in 2011 and 17.6% in 2021 [21], and this number is anticipated to reach approximately 23.8% by 2030 [22]. To address this demographic shift, the Singapore government has been constantly exploring technological solutions and launching health initiatives to proactively meet the older population’s health care needs. Some examples include HealthHub—the 1-stop *digital health care companion* to manage appointments and access personal medical records [23] and National Steps Challenge—the *world’s first population-level and fitness tracker–based physical activity* that encourages Singapore residents to track their daily moderate to vigorous physical activities and get rewards [24]. Despite these efforts, there remains a scarcity of studies into the patterns of the older population’s HSB and the potential of technologies in facilitating their health seeking and addressing their health needs.

### Aim and Objectives

This study aimed to investigate HSB and associated technology use among older people in the Singapore community. We hoped to gain deep insights into how this population makes decisions when they engage with the health system and use technologies; identify any unmet needs; suggest ways in which technology can be leveraged to address these needs; and ultimately, propose recommendations for practical strategies that ensure they are not excluded from the efforts to build the *smart nation*. In particular, we would like to answer the following research questions:

1. What are the activities of HSB (consequences) in the context of aging in Singapore?
2. How have technologies been incorporated into older people’s health seeking?
3. What considerations do older adults take into account when choosing technologies to meet their health needs?
4. What are the implications for practice?

## Methods

This paper presents partial data from a large study that explored the potential of telehealth in addressing unmet health needs and the attitudes toward telehealth among older individuals in the Singapore community.

### Study Design

We used a phenomenological approach to explore the lived experiences of older adults’ HSB and associated technology use. The reporting of this study was guided by COREQ (Consolidated Criteria for Reporting Qualitative Research), a 32-item checklist [25].

### Participant Recruitment

Inclusion criteria were (1) being ≥50 years old, (2) being Singapore citizens or foreigners who are dwelling in the Singapore community in the long term, and (3) being able to read and converse in English or Mandarin.

The study was planned during the *stabilization phase* [26,27] (with heightened COVID-19 safety management measures [SMMs] to slow down the rate of transmission), and participant recruitment started in January 2022, during the *transition phase* in Singapore (groups of up to 5 people were allowed in social gatherings) [28]. We aimed to recruit a diverse range of participants in terms of sociodemographic and socioeconomic characteristics such as age groups, ethnicities, education backgrounds, occupations, income levels, and housing types. To achieve this, we adopted convenience sampling with various recruitment strategies, considering the unpredictable SMMs imposed owing to the COVID-19 pandemic. Before data collection, we posted our study posters on social media platforms (Facebook and Instagram) and shared our study information with some chat groups for older adults. We then obtained referrals from our early participants and stopped recruitment until no new themes were identified. Subsequently, we reviewed the profiles of the interviewees and purposively recruited more participants of interest from the community centers and public areas during the *COVID-19–resilient nation phase* (the social distancing measures were relaxed, and the level of the Disease Outbreak Response System Condition was adjusted to yellow) [29]. We ceased the whole participant recruitment process in July 2022, as no new insights were obtained (thematic data saturation was reached).

### Data Collection Tools

This qualitative study used 2 data collection tools—a web-based registration form and a semistructured interview guide.

#### Web-Based Registration Form

The registration form was used to collect the participants’ sociodemographic and socioeconomic characteristics, existing health conditions, ownership of mobile devices, access to Wi-Fi, and consent to participate in the subsequent interview.

#### Semistructured Interview Guide

**Figure 1:**
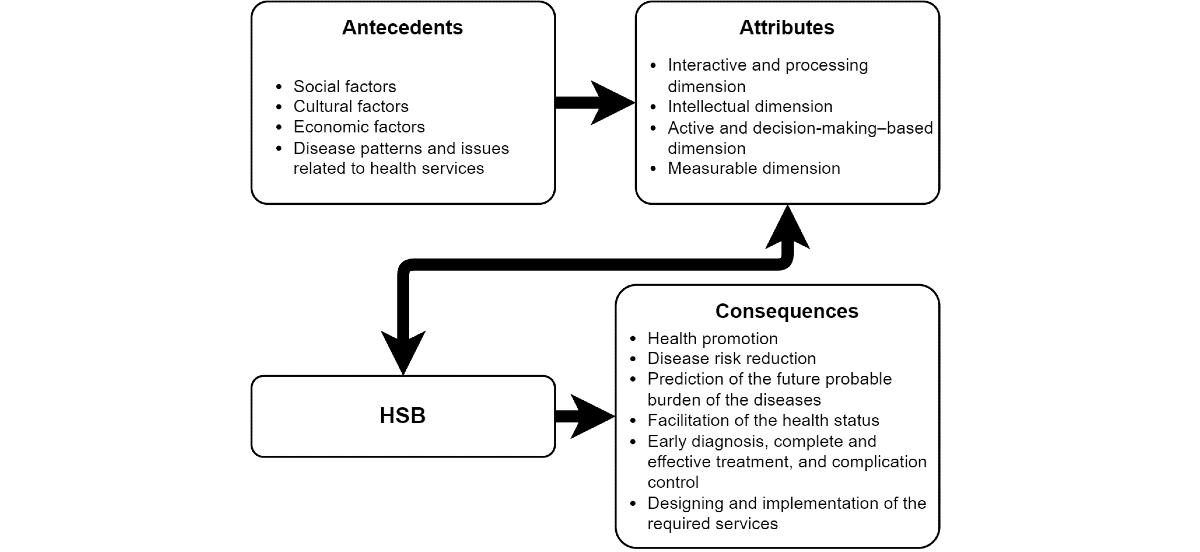
The health-seeking behavior (HSB) model by Poortaghi et al [30].

The preapproved interview guide was developed by YZ and reviewed by WPT. In our study, we adopted the definition by Chinn and Kramer [9] and the evolutionary content analysis of HSB by Poortaghi et al [30] in the nursing setting. According to Poortaghi et al [30] HSB has 4 crucial attributes, namely, interactive and processing dimension, intellectual dimension, active and decision-making–based dimension, and measurable dimension (Figure 1). That is, HSB is an ongoing process involving a logical sequence from the symptom evaluation to the decision of using different care, the individual’s efforts to pursue an acceptable level of well-being, the approach to acquiring health information, and the routine of constant health monitoring and behavior change to move toward high-level wellness [30]. The detailed list of guiding questions for the semistructured interviews can be found in Multimedia Appendix 1.

### Data Collection Procedure

Data collection occurred from April 2022 to July 2022. Older adults who expressed interest in participating were invited to complete the web-based registration form and screened to check their eligibility for the interview. Eligible participants were then informed about the data collection procedure; recording of the interviews; and how the data would be analyzed, reported, and protected through written informed consent. Only those who gave their consent were scheduled for an interview. To comply with the SMMs, Zoom (Zoom Video Communications Inc) was chosen as the default interview medium, and a manual was provided to the participants whenever needed. Participants who had unresolvable difficulty with Zoom were offered the option of a face-to-face interview. YZ contacted all eligible candidates either via phone calls or WhatsApp to explain the study details again and scheduled the interviews at their convenience. Written informed consent and permission for interview recording were obtained in advance. Each participant was only required to attend 1 interview session. All interviews were conducted on a one-on-one basis and were video recorded with participants’ consent.

At the beginning of each interview, the study details were explained again, and verbal consent was obtained and recorded. YZ conducted all the interviews. She documented her observations through field notes and confirmed important findings with the participants during the interviews to ensure accurate interpretations. The interview consisted of 3 sections: HSB, associated technology use, and how the participants selected technologies. Participants received shopping vouchers worth SG $20 (US $15) upon successful completion of the semistructured interview.

### Data Analysis

Data analysis was performed after each interview. Each participant was assigned a unique identifier before data analysis, and all study data were deidentified during the transcription process. YZ manually transcribed all interviews verbatim in Word (Microsoft Corp) and transferred all text data to Excel (Microsoft Corp) for thematic analysis. Participants who preferred Mandarin had their transcripts translated by YZ and reviewed by WPT, to ensure the highest level of accuracy. The individual was chosen as the unit of analysis to identify the patterns of HSB and technology use in the older population. Next, YZ conducted inductive thematic analysis, in which a line-by-line coding approach was used while reading through the qualitative data. The codes were then categorized based on the research questions, and the generated pattern codes were used to identify themes. YZ and WPT met regularly to discuss the codes and themes until consensuses were reached, to enhance the validity of the analysis.

### Ethics Approval

This paper presents partial data from a large study, which has been approved by the institutional review board of Nanyang Technological University Singapore (reference IRB-2021-797).

## Results

### Participant Recruitment and Characteristics

In the period from February 2022 to April 2022, 12 individuals signed up for our study after seeing our study poster on social media or in the chat groups for older adults, but only 2 (17%) met our inclusion criteria. Additional 8 participants were then recruited via the referral of the initial 2 eligible participants until no further new themes were generated. We subsequently visited different community centers and public areas and recruited 5 participants with low socioeconomic status or from other ethnicities before we reached thematic saturation. In total, we conducted 13 Zoom interviews where participants remained in their homes, and 2 face-to-face interviews were conducted at the community centers in close proximity to the participants’ residences. The mean duration of the interviews in the larger study was 39 minutes (SD 15 minutes 26 seconds).

The 15 participants were aged between 55 and 73 (mean 65.8, SD 6) years, among whom 9 (60%) were women, 13 (87%) were Chinese, 6 (40%) were still working, and 8 (53%) had a personal monthly income of <SG $1000 (<US $752). All participants (15/15, 100%) owned a personal smartphone, but 13% (2/15) of them did not have Wi-Fi access at home. Their characteristics are summarized in Table 1, and detailed information can be found in Multimedia Appendix 2.

**Table 1:** Summary of participants’ characteristics (N=15).

Characteristics			Values, n (%)
**Age range (years)^a^**			
	55-60	2 (13)	
	60-70	8 (53)	
	70-80	5 (33)	
**Sex**			
	Female	9 (60)	
	Male	6 (40)	
**Ethnicity**			
	Chinese	13 (87)	
	Indian	1 (7)	
	Malay	1 (7)	
**Number of known health conditions**			
	0	7 (47)	
	1	3 (20)	
	2	4 (27)	
	3	1 (7)	
**Highest education achieved**			
	Primary and secondary	5 (33)	
	Preuniversity	4 (27)	
	Degree and postgraduation	6 (40)	
**Employment status**			
	Homemaker	3 (20)	
	Retired	6 (40)	
	Employed part-time	4 (27)	
	Employed full-time	2 (13)	
**Estimated current personal monthly income, SG $ (US $)**			
	0 (0)	5 (33)	
	<1000 (<752)	3 (20)	
	1000-1999 (752-1503)	1 (7)	
	2000-2999 (1504-2255)	2 (13)	
	3000-3999 (2255-3006)	1 (7)	
	4000-4999 (3007-3758)	2 (13)	
	7000-7999 (5263-6014)	1 (7)	
**Housing type**			
	Rental flat^b^	1 (7)	
	4-room or 5-room HDB^c^ flat^d^	4 (27)	
	Terrace house^e^	1 (7)	
	Semidetached house^f^	2 (13)	
	Condominium^g^ (excluding executive condominium^h^)	7 (47)	
**Ownership of a smart device**			
	Smartphone	15 (100)	
	Tablet	7 (47)	
	Personal laptop (not for work purposes)	11 (73)	
**Access to Wi-Fi at home**			
	Yes	13 (87)	
	No	2 (13)	

^a^Mean age 65.8, SD 6 years.

^b^An affordable housing rental option for Singaporeans with low income [31].

^c^HDB: Housing and Development Board.

^d^A public housing type in Singapore, which is affordable and can be easily purchased by the average Singaporean as they are subsidized by the government and are offered with housing grants (4-room HDB flat: 3 bedrooms and 1 living room; 5-room HDB flat: 4 bedrooms and 1 living room) [32].

^e^A dwelling house with its own land title that forms part of a row of at least 3 dwelling houses abutting the common boundary party walls [33].

^f^A single-family duplex dwelling house that shares a common wall with the next house [34].

^g^An apartment that is part of a development not managed by the HDB, owned by unit owners who share common areas and facilities with other unit owners within the development [35].

^h^A strata-titled property, with comparable designs and facilities to other condominiums [35].

### Consequences of HSB in the Aging Context

#### Overview

Our interviews identified five main consequences of HSB, namely, (1) health information seeking, (2) health maintenance, (3) early diagnosis and complication control, (4) responses to symptoms, and (5) health service use.

#### Health Information Seeking

##### Health Education Participation

In total, 87% (13/15) of the participants have attended government, hospital, university, and other community health educational sessions or self-read the health information on mobile health (mHealth) apps, through which they could gain knowledge about various health topics:

I’m a volunteer with HPB, and we do conduct health talks. I’m interested in that and I’m still active with it. They have [the] War of Diabetes, [and] some [talks] focus on exercise and nutrition.

Participant 2

I attended the wellness and health [talk] held by Boon Lay CC last year on the 26th of December, the day after Christmas. I registered myself there to study health and wellness and they taught me to download some of the apps such as 365 [the Healthy 365 app]. I attended the health coaching course offered by NTU [Nanyang Technological University].

Participant 13

I attended one group education in KTPH [the Khoo Teck Puat Hospital].

Participant 15

Overall, 5 factors were identified to have an impact on the preference and judgment of health education, namely, the instructors’ qualification, instructors’ language or terminologies, usefulness and relevance of the content, cost, and personal interest. Sample responses are summarized in Multimedia Appendix 3.

##### Health Information Searching and Sharing

In total, 87% (13/15) of the participants actively searched for health information, and the most common information sources were the web (eg, Google, Wiki, and forums), mHealth apps, social networks (family members and friend groups), health professionals, and their fields of medicine expertise:

Yes, I will check online or ask friends...I [also] use the HealthHub app...I found it very useful. And it does have family program[s] and health tips that I can go to find out more.

Participant 2

Normally my friends and I will share health information in [the] WhatsApp group, we are in a retiree group.

Participant 6

I collect health information from the doctor.

Participant 8

Actually, I know all these investigations [that have been] done quite well because I worked in the hospital before, so I know all these tests, what they are for, and you must take some medication regularly.

Participant 10

##### Health Information Evaluation

Participants adopted multiple methods to evaluate the credibility of the health information: (1) relying solely on official sources, (2) seeking verification from health professionals, (3) checking the credibility of information sources, (4) searching for scientific evidence, (5) gathering information from individuals with similar experiences, and (6) self-experiment. Sample responses are summarized in Multimedia Appendix 4.

Participants shared that their health information searching and participation in health education could empower themselves with health-related knowledge. They have gained a deep understanding of well-being and diseases, and that has led them to better awareness of their bodies and a positive shift in their health behavior:

I become aware of the diseases, and for example, I know that if I eat a lot of fatty stuff, my cholesterol will be affected, thereby when I choose the food I will read the label, so in my daily life in my choice of food and all those, I become educated. After I know all the knowledge but don’t practice in my daily life, it’s like zì jǐ zhǎo sǐ [killing myself] right?

Participant 5

We all understand that exercise is good for our health which I’m doing every day. Moderation is the key for any food you take, and I never take excessive [but] just moderate and take what I need. And also need to keep your mental health good, and the more you use your brain the more you can keep your brain; [if] you don’t use your brain, you may get dementia. Human beings need group activities like we walk together, and we go to the SACs [Senior Activity Centers] to talk to the elderly. And this way you need friends to be able to listen to your problems, and you don’t separate from the society, and that might lead to some mental problems like depression and dementia.

Participant 6

#### Health Maintenance

All participants (15/15, 100%) shared that they have been pursuing healthy lifestyles and strengthening social connections to maintain their physical, mental, and social capacities:

Enough sleep, proper diet, [and] regular exercise [help me maintain my health]. [I’m] getting myself active in daily activities, just to keep me mentally and physically engaged.

Participant 3

I have several groups like my line dancing group, volunteering groups of a few institutions...These people really keep me mentally on the spot.

Participant 5

#### Early Diagnosis and Complication Control

##### Health Screening and Follow-up Care

All participants (15/15, 100%) have been undergoing regular health screenings and follow-up care, with frequency varying from several months to several years, contingent on the purposes (eg, disease screening and chronic condition monitoring):

Once every three or four months I go for my health screening, once a year I get my flu injection, [and] once a year I also go [for] ultrasounds on my lungs, chest, and prostate.

Participant 8

##### Self-measurement of Vital Signs

Vital signs are noninvasive objective measures of a person’s physiological function using simple equipment, such as pulse, temperature, blood pressure, and respiratory rate [36]. They could serve as a basic means to communicate about a person’s health status and keep track of both acute and chronic conditions [36]. In our study, only 7% (1/15) of the participants reverently measured his vitals at home and 60% (9/15) of the participants determined when to perform self-measurement based on the bodily symptoms or daily activities, whereas the rest did not practice self-measurement. Overall, four reasons were reported: (1) self-perceived good health, (2) inability to schedule, (3) lack of necessary equipment at hand, and (4) difficulty in obtaining accurate measurements. Sample responses are summarized in Multimedia Appendix 5.

#### Self-perceived Health and Responses to Symptoms

Despite the impacts of the COVID-19 pandemic on the participants’ physical, mental, and social well-being (as seen in Multimedia Appendix 6), all participants (15/15, 100%) perceived their health to be good to excellent and stated that they rarely fell sick. In the event of any symptoms, three different actions were taken: (1) self-treatment; (2) seeking prompt professional medical attention; and (3) undergoing a logical process from self-treatment to seeking professional medical attention, depending on the severity of symptoms. Sample responses are summarized in Multimedia Appendix 7.

#### Health Service Use

##### Satisfaction With the Living Environment

All participants (15/15, 100%) were satisfied with their living and surrounding environments. They could easily access recreational spaces (eg, fitness facilities, parks and open spaces, and walking and cycling paths), amenities (eg, grocery stores and health stores such as Guardian), public transportation (eg, bus stops and Mass Rapid Transit stations), and health care services (eg, outpatient polyclinics, private general practitioners [GPs], and hospitals), and this could make their life more convenient and healthy:

My house is very accessible, and during COVID time we have walked to all the connectors, and we can also walk around within the whole estate.

Participant 7

Good! It’s within [a] 10-20 mins walk to the facilities. Every day I make the trip down to the mall with a library there and use the shopping mall and polyclinic. I also walk, it’s like a form of exercise.

Participant 10

##### Access—Cost

All participants (15/15, 100%) shared that various government subsidies, such as the Community Health Assist Scheme, Merdeka Generation Package, and Pioneer Generation Package, enabled them to access affordable medical services:

We have the CHAS [Community Health Assist Scheme] cards, we have the Pioneer Generation card, so the charge is minimal, I will say [it is] very affordable...[The health screening is] very affordable, there are two layers of subsidies.

Participant 10

##### Access—Waiting Time

Most participants (11/15, 73%) reported that physicians’ consultations were fast, but the waiting time could be hours, even with an appointment:

The consultation is very fast, but the waiting time to see the doctors can take hours.

Participant 1

My appointment was set at 11:30 [am] in the morning [after a long weekend], but I did not see the doctor until 4:30 pm, that is unusual, and even the doctor said "today is unusual". The consultations are usually very fast.

Participant 6

##### Interaction—Communication With Health Professionals

Our participants reported a general desire to engage in more health-related discussions with their physicians, but the physician-patient communications varied with health professionals and could affect patients’ emotions:

Very good because we have been seeing this family doctor for many many years, so we are very comfortable. The communication is very good.

Participant 7

I hardly see GP so I cannot comment on it. I only can comment [on] those doctors that I have seen in [the] polyclinic. These doctors keep on changing, it’s not a specific doctor. I find it poor because sometimes I do feel that they are just doing their job only. Poor in the sense that I feel that sometimes they just do their job to move you away because they have so many patients. But sometimes there are some pretty good doctors, and they do try to explain, so I cannot generalize that. I will see 2 extremes: when I meet good doctors, I’m very happy, but when I meet these so-called poor doctors who are not with the patient, then I will say “okay lah I go polyclinic what do you expect.”

Participant 9

### Technology Use in Older Adults’ Health Seeking

**Figure 2:**
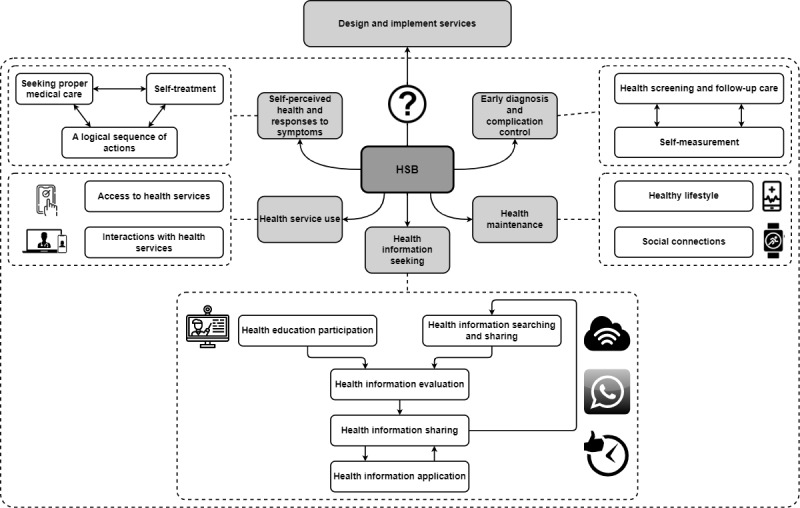
Summary of the use of technologies in older adults’ health seeking. HSB: health-seeking behavior.

We explored the use of technologies in each consequence of the older adults’ HSB (Figure 2) and extracted 4 main themes: theme 1—the most widely used digital technologies among the older population are mHealth apps and wearable devices with associated wellness programs launched by government agencies and local companies, and these technologies have the potential to enhance health communication, promote health maintenance, and increase access to health services; theme 2—information communication technologies (ICTs) and social media, although not primarily designed for health purposes, play a substantial role in easing the process of seeking health information and managing symptoms; theme 3—although the outbreak of the COVID-19 pandemic has resulted in some alterations to older adults’ well-being, it has catalyzed the adoption of telehealth as a complement to access health care services; and theme 4—older adults have different considerations when selecting technologies to facilitate their health seeking and fulfill their health needs.

#### Theme 1—The Most Widely Used Digital Technologies in Health Seeking Among the Older Population

##### Health Communication

Health communication leverages technological innovations, such as mass media and multimedia to spread health information, enhance public understanding of health, educate the public about health concerns, and keep critical health issues on the public agenda [37]. Our participants shared that they were able to locate information about health programs and health tips through mHealth apps developed by the government:

I use the HealthHub app...I found it very useful. And it does have family program[s] and health tips that I can go to find out more.

Participant 2

##### Health Maintenance

Most of the participants (10/15, 67%) reported using various mHealth apps and wearable fitness trackers, launched by either government or local companies, to track their daily physical activities and stay motivated toward achieving their health goals. They could also receive attractive incentives from the associated health campaigns:

I use it [Healthy 365 app] for step tracking. I participated [in] the [National] Steps Challenge as this is free money...I call it preventive medicine because they pay you to lose weight.

Participant 9

I use [the] SingTel StepUp program also, every month I can redeem at least 2 free Gigabytes for myself.

Participant 14

##### Overcoming Challenges in Health Service Use

Although most of the participants (11/15, 73%) expressed frustration with the long waiting time to see physicians, a few of them shared that mHealth apps such as the HealthHub app could offer them a platform to receive real-time updates about the waiting time and manage their nonurgent appointment bookings:

With the appointment on the HealthHub app, I can check how many more patients [are] ahead, then I hang out outside. It doesn’t bother me if I have to wait very long.

Participant 5

I was using HealthHub to check the information about my appointment.

Participant 14

Besides managing appointments, participants said that they could also view test results and health reports on the mHealth apps:

I go there [the HealthHub app] to do [medical appointment] booking, checking my appointment, and checking my blood test results.

Participant 9

#### Theme 2—Application of ICTs and Social Media in Health Seeking

##### Health Information Seeking

Apart from health professionals, ICTs and social media were also the active sources of health information seeking and sharing for 87% (13/15) of the participants:

Google and read the review of the medicine, then go to some forum. I will check as many sources as I can.

Participant 5

Sometimes I check Facebook and YouTube.

Participant 12

From time to time we get [health information] from friends, we get the WhatsApp message to say “hey this is good, you can try this exercise...”

Participant 8

##### Responses to Symptoms

Among the participants who opted for self-treatment as their initial response to symptoms, web-based resources and peer sharing through ICT could provide them with relevant health information, especially from friends who have experienced similar symptoms:

Sometimes [I] can see [health information] from YouTube, like you press this pressure point, you massage this area, and your BP will be reduced; or you take some kind of herbal soup. Information is everywhere, so it depends on how you want to capture it. I check this info on YouTube and Facebook.

Participant 14

Sometimes, like when I had COVID, I shared with them [my friends in the WhatsApp group] the experience of the first few hours of having it, [how I felt after] three days of feeling about it and how I did the ART test, and the results. So it’s the sharing of experiences and exchanging thoughts about what happened, are we [we are] facing the same issues or different [ones].

Participant 3

#### Theme 3—Telehealth Adoption in Health Seeking

##### Telemedicine or Teleconsultation

During the COVID-19 pandemic, telemedicine served as an alternative way to access health care services. A few participants have tried teleconsultation when they were tested positive for COVID-19 and had to self-isolate:

I tried to do a virtual or teleconsult[ation] when I self-tested COVID...I talked to the app on my mobile phone, and I called in to schedule an appointment with the doctor. After the consult[ation], they sent the mediation in within 3 to 4 hours. The consultation, medication, and delivery were very reasonable, and it’s cheaper than going to a GP.

Participant 3

##### Tele-Education

Several participants (5/15, 33%) shared that some of their regular health education sessions were adapted to a web-based format via Zoom amid the COVID-19 pandemic, and this allowed them to continue their learning journey with tele-education:

We had quite a number of Zoom lectures from HPB [the Health Promotion Board].

Participant 1

365 [Cancer Prevention Society] sometimes has little talks about nutrition, I do go in and listen on Zoom.

Participant 9

##### Remote Health Tests

The COVID-19 pandemic did not eradicate the participants’ regular health screenings, but the initial lockdown has caused some delays. Some participants chose to postpone their health screening to prevent possible infection and were offered self-tests as a substitute:

I didn’t go for the blood test as my husband [who is a doctor] told me not to go. But I still did one free test which they sent to me, I collected the specimen and sent it back to them.

Participant 5

#### Theme 4—Considerations When Selecting Technologies to Facilitate Health Seeking

When asked about how to select technologies, eight factors were reported by the participants, namely, (1) perceived usefulness, (2) comprehensiveness of features, (3) perceived ease of use, (4) performance and quality, (5) recommendation by the social network, (6) cost, (7) rewards, and (8) credibility. Sample responses are summarized in Multimedia Appendix 8.

#### Archetypes

The participants in our study exhibited a relatively consistent response pattern. However, our results, coupled with insights from our participants’ observations within their social networks, have led us to propose 4 archetypes. These archetypes were designed based on factors such as the activeness level of their HSB, access to technologies, willingness to adopt technologies, and capability to use technologies (Textbox 1).

**The 4 proposed archetypes.**

- Archetype 1
  Inactive in health seeking
- Archetype 2
  Proactive in health seeking but hesitant to adopt technologies
- Archetype 3
  Proactive in health seeking and receptive to technologies but facing challenges in accessing or using technologies
- Archetype 4
  Proactive in health seeking, receptive to technologies, and able to access and use technologies

## Discussion

### Principal Findings

This study expanded upon the literature by adopting the multidimensional model by Poortaghi et al [30] to explore the HSB of older adults in the Singapore community. Unlike previous studies that only focused on health system use and illness responses, our study explored older adults’ health-seeking practices with a comprehensive approach, including health information seeking, health maintenance, early diagnosis and complication control, responses to symptoms, and access to and interaction with health care services. We also examined older adults’ technology use and considerations for technology selection, and our results offered a different perspective from a previous qualitative study in Singapore, which found that older adults’ HSB was technology independent [38]. Our findings suggested that technologies have been seamlessly integrated into older adults’ health-seeking activities and have a promising capability of encouraging proactive HSB.

### Consequences of HSB in the Aging Context

We identified a total of 5 major consequences of HSB, namely health information seeking, health maintenance, early diagnosis and complication control, responses to symptoms, and health service access and interaction, which are consistent with the model by Poortaghi et al [30]. Although we made an effort to recruit participants with varying sociodemographic and socioeconomic characteristics and observed relatively consistent patterns, the exploratory nature and limited sample size of qualitative research may restrict the generalizability of our findings. Future studies could expand on our study by incorporating the antecedents in the model by Poortaghi et al [30] and evaluating the impact of social, cultural, economic, and health-related factors on HSB, to uncover the unfulfilled needs of the older population.

### Technology Use in Older Adults’ Health Seeking

A key discovery was the older adults’ wide engagement with technologies and associated health campaigns launched by the government and local companies. Supporting the findings by Low et al [38], most of our participants (10/15, 67%) were motivated to use wearable fitness trackers together with mHealth apps to keep track of their physical activities. These technologies could provide users with real-time feedback and motivate them to achieve the predetermined lifestyle goals [39]. Moreover, the financial incentives offered by these initiatives seemed to be an important motivator for older people to use such technologies and encourage positive changes and discourage negative ones in health behavior [40]. Future studies could further investigate the extent to which technology-driven behavior change techniques such as incentive schemes can alter users’ behavior and improve their health outcomes. It would also be valuable to explore how such techniques can be integrated with policy tools to facilitate sustainable behavior change.

Technologies offer a solution to overcome the challenges in health service use and interaction. Although all our participants (15/15, 100%) were satisfied with their living and surrounding environments, were able to easily access primary care, and could enjoy affordable medical services through various government subsidies, they pointed out 2 challenges during the interaction with the current health systems. Similar to a theme in the findings by Lee et al [5], many of our participants (11/15, 73%) also shared their experience of waiting several hours for just a few-minute consultation, particularly after public holidays and during the COVID-19 pandemic. In addition, they also encountered communication barriers and even felt discouraged from engaging in further communication as they felt *pushed away* by their health professionals. These might be attributed to a shortage of health professionals in the country, as reported by the Ministry of Health that the physician-to-population ratio was 1:399 and there were only 2.5 physicians per 1000 population in 2019 [41]. In addition to incorporating self-service medical booths [42], the use of digital technologies such as the machine learning–based solution being developed by the Singapore National Eye Center, called Appointment Scheduling Optimizer, could reduce patients’ waiting time [43]. Telehealth, which has the potential to reduce expenses and time spent on traveling and waiting [44], might offer another viable solution, but future studies are needed to further reaffirm the acceptability, effectiveness, and cost benefits of telehealth services.

ICT and social media expand people’s alternatives in searching for health-related information; however, they are not tailored to health intentions and cannot alleviate, diagnose, or cure diseases. In contrast to the findings by Lin et al [45] that only a small portion of the participants searched for health information on the web using their mobile phone, a high percentage of older participants in our study used their smartphones for searching and sharing health information, via web-based resources, social media, mHealth apps, and messaging through social network communication tools. This difference may stem from the improved accessibility and easy sharing of information through mobile phones in recent years. Some of the government initiatives have also begun to harness these platforms, such as HPB’s posting of health-related videos on YouTube for health promotion [46] and the launch of the official COVID-19 channels on Telegram and WhatsApp to broadcast news updates, deliver important announcements, and reduce the spread of misinformation during the COVID-19 pandemic [47]. An unexpected result was that almost all participants (14/15, 93%) made an effort to question and assess the credibility of the health information. They exhibited skepticism toward the health information encountered and used various means to verify its credibility. Our findings suggested that technologies have shaped health information seeking as a collective and collaborative effort—people first gather information; next, verify and circulate it through their social network; and then, apply it in their daily practice and provide feedback. Through this cyclic seeking-verifying-sharing-applying-feedback process, they can reaffirm the credibility and effectiveness of the information through firsthand experience.

Although the COVID-19 pandemic has brought some alterations to older people’s well-being and disruptions to their lifestyle practices, it also catalyzed technological innovation and adoption. For example, telehealth, although not a novel concept [48,49], gained increasing attention amid the COVID-19 pandemic [50]. Our participants shared that different telehealth modalities could enable them to continue their access to health care services and health education. Future studies could further evaluate the role of telehealth in aging care.

### Implications for Practice

This section serves as the last consequence in the HSB model by Poortaghi et al [30]—*design and implement needed services*.

#### Health Communication and Promotion

According to our findings, ICT and social media appeared to be promising channels for obtaining and exchanging health information. Previous studies also reported that many older citizens in the Singapore community are not resistant to technologies, and they spent more time on smartphones watching dramas, playing games, and chatting on messaging platforms [51,52]. Leveraging these platforms and launching eHealth communication and promotion campaigns could be a viable strategy to increase health awareness and encourage participation among the older population.

#### Health Education

Our participants shared that the overriding factors affecting their preference and evaluation of health education are the qualifications of and language used by the health instructors, usefulness and relevance of the content, cost, and personal interest. Although there is no *one-size-fits-all* solution, future health education programs could take these factors into consideration.

#### Technology Design, Improvement, and Implementation

Participants shared various factors in technology selection, but the results may be limited by the types of such technologies that they have used before. In contrast, this also implies that older adults might be granted access to a wider variety of digital technologies. Besides perceived usefulness, perceived ease of use, and financial cost found by Lin et al [45], our participants also shared that the comprehensiveness of features, quality of performance, recommendations by their social network, rewards, and credibility are the other overriding considerations. Taking these factors into consideration would be useful not only for technology developers when enhancing current technologies or creating novel ones to promote better user-centered designs but also for policy makers in the process of digital transformation and building the *smart nation*.

#### Clinical or Community-Based Telemonitoring Services to Support Older Adults’ Self-measurement Practice

A possible gap we identified in the participants’ HSB was a lack of self-measurement of their vitals, especially for those with chronic conditions. Single-point vital sign measurements have shown to be less sensitive in detecting disease processes as a result of the diverse but individual age-related physiological changes and comorbidities, whereas successive or serial measurements may enhance the sensitivity in detecting disease processes, especially when viewed in conjunction with individualized reference ranges [36]. In Singapore, some polyclinics have introduced telemonitoring services for patients with chronic health conditions, and such services have been well accepted [53,54]. Patients who rely solely on physical examinations, who do not own a personal device, and who are occupied with daily chores can get the necessary devices and benefit from such services for chronic disease management, as prompt measurements can be taken beyond the health care setting. Besides clinical approaches, community-based initiatives can be used to make technology more accessible to the public and aid those who have difficulty in obtaining accurate measurements. For example, telemonitoring kiosks with trained health ambassadors in the community can be set up to provide users with health tips and gather data to help health professionals make better decisions. By implementing these ground-up strategies, older individuals can enjoy better access to quality health care services and technologies; reduce expenses associated with travel and medical equipment purchases; take a more proactive role in managing their health; and ultimately, reduce health disparities. Future studies could further investigate the effectiveness of telemonitoring in detecting diseases and reducing complications, hospital readmissions, and mortality rates in aging care.

#### Possible Solutions to Facilitate the Health Seeking of the Proposed Archetypes

It is important to note that although technologies play a promising role in assisting older people’s health seeking, they are not mandatory. People may have various reasons for using or not using technologies; therefore, it is crucial to uncover their specific health needs and ensure that no one is forced to use technologies or left behind in digital inclusion.

##### Archetype 1—Inactive in Health Seeking

For archetype 1, the priority might be identifying older people’s unfulfilled needs and seeking tangible resources from both health care and community settings. Some possible strategies include educating them about the significance of proactive HSB through public health campaigns, providing regular updates about their health status, and ensuring that they are not left behind in digital inclusion.

##### Archetype 2—Proactive in Health Seeking but Hesitant to Adopt Technologies

For archetype 2, it might be helpful to work with health professionals and assess the need for incorporating technologies in their health seeking. Through this joint effort, they can determine the most suitable solutions to meet their specific needs. At the same time, they can be invited to attend health events or participate in awareness campaigns to gain firsthand experience regarding the benefits of technologies and make informed decisions.

##### Archetype 3—Proactive in Health Seeking and Receptive to Technologies but Facing Challenges in Either Accessing or Using Technologies

For archetype 3, the focus could be on lowering the barriers to using technologies. Some possible strategies might be introducing simple technologies in small steps, offering assistance and support to build people’s technology skills (eg, the *Senior Go Digital* program [55]), encouraging social engagement and creating a supportive community, and improving both user-friendliness and accessibility of technologies for the older population.

##### Archetype 4—Proactive in Health Seeking, Receptive to Technologies, and Able to Access and Use Technologies

Archetype 4 might be the most suitable candidate for technology use and require the least amount of support. The focus could be on developing innovative technologies and policy solutions to sustain and enhance their HSB.

### Strengths, Limitations, and Future Studies

This study had several strengths. Conceptually, we extended the literature by adopting a multidimensional model and assessed the older adults’ HSB using a more comprehensive approach, and our findings further reiterated this model. Methodologically, we adopted multiple recruitment strategies to reduce selection bias, and we managed to get insights from older adults with varying sociodemographic and socioeconomic characteristics in Singapore. Despite these strengths, we acknowledge that our study also had some limitations. First, most participants (13/15, 87%) were Chinese, which might be because of the inclusion of only English and Mandarin speakers, considering the researchers’ language proficiency. Future studies could be extended to Malay, Tamil, and Chinese dialect speakers. Second, owing to the COVID-19 pandemic restrictions, the main method of participant recruitment and contact was through social media and WhatsApp, which could have resulted in a possible bias toward individuals who were more technologically advanced and had better access to technologies. Future studies should attempt to reach out to individuals who are digitally illiterate or homebound. Finally, this was an exploratory qualitative study with a limited sample size, and the choice of the individual as the unit of analysis to understand group phenomena may overlook certain fine points and variations among individuals. Large-scale quantitative or mixed methods studies could further investigate the impact of social, cultural, economic, and health factors (the antecedents in the model by Poortaghi et al [30]) on older adults’ HSB and how technologies can address these inequalities.

### Conclusions

In conclusion, we have extended the literature and investigated older adults’ HSB and associated technology use with a more comprehensive approach. Unlike the commonly held belief that older adults resist technologies and lack technological proficiency, our results showed that technologies could play a promising role in facilitating older adults’ health seeking. Our findings have implications for the design and implementation of health services and policies.

## Appendix

Multimedia Appendix 1 Semistructured interview guide.

Multimedia Appendix 2 Detailed participants' characteristics.

Multimedia Appendix 3 Sample responses regarding the preference and evaluation of health education.

Multimedia Appendix 4 Sample responses regarding evaluating the reliability of information sources.

Multimedia Appendix 5 Sample responses regarding self-measurement.

Multimedia Appendix 6 Sample responses regarding the impact of the COVID-19 pandemic on older people’s perceived health.

Multimedia Appendix 7 Sample responses regarding health service use.

Multimedia Appendix 8 Sample responses regarding the choice of health technologies.

## Abbreviations

COREQ: Consolidated Criteria for Reporting Qualitative Research
GP: general practitioner
HSB: health-seeking behavior
ICT: information communication technology
mHealth: mobile health
SMM: safety management measure

## Data Availability

The data sets generated and analyzed during this study are available from the corresponding author upon reasonable request.
