# Supplementary material for: Health-Seeking Behavior and Its Associated Technology Use: Interview Study Among Community-Dwelling Older Adults
Source: JMIR Aging. 2023 May 4;6:e43709. doi: 10.2196/43709 (PMC10196894; doi:10.2196/43709)
Supplement: Multimedia Appendix 1 [file aging_v6i1e43709_app1.docx]

Multimedia Appendix 1. Semi-structured interview guide

| Dimension | Interview questions |
| --- | --- |
| Intellectual dimension | 1. How do you rate your overall health status? 2. How do you maintain your physical and mental health? 3. How often do you go for regular health screenings? 4. What were some changes in your health status, health routine, and health screening during the COVID-19 pandemic? |
| Interactional and processing dimension | 1. What do you do when you feel unwell? 2. How do you feel about the environment of and around your place? 3. How far is the polyclinic/GP that you usually visit away from your place? 4. In general, how long do you spend waiting for seeing the doctors? What about the time spent inside the consultation rooms? 5. How much are you charged by vising polyclinic/GP (e.g., disease treatment, health screening)? 6. Are you keen to know more about your health and talk to the doctors? 7. How do you find communication with health professionals? |
| Active and decision-making-based dimension | 1. How do you look for health information? 2. How do you trust the health information that you find? 3. How do you follow the advice/tips given by these resources? 4. Did you attend any health education? How do you evaluate it/them? 5. Can you give some examples of how you practiced what you learnt in health education in your daily life? |
| Digital technology utilization | 1. What are the purposes of using digital tools/health apps, if any? 2. How do you choose digital tools, given that there are various such applications in the market? |
