# Supplementary material for: Health-Seeking Behavior and Its Associated Technology Use: Interview Study Among Community-Dwelling Older Adults
Source: JMIR Aging. 2023 May 4;6:e43709. doi: 10.2196/43709 (PMC10196894; doi:10.2196/43709)
Supplement: Multimedia Appendix 2 [file aging_v6i1e43709_app2.docx]

Multimedia Appendix 2. Detailed participants' characteristics

| Participant UID | Age (years) | Sex | Ethnicity | Highest education achieved | Employment status | Estimated monthly income (in Singapore dollars) | Housing type | # of known health conditions | Ownership of a smart device | Access to Wi-Fi at home |
| --- | --- | --- | --- | --- | --- | --- | --- | --- | --- | --- |
| EP01 | 73 | Female | Indian | Degree | Homemaker | 0 | Condominium (excluding EC) | 1 | Smartphone, Tablet | Yes |
| EP02 | 65 | Female | Chinese | O-Level | Homemaker | 0 | Condominium (excluding EC) | 0 | Smartphone, Tablet | Yes |
| EP03 | 64 | Male | Chinese | Postgraduate | Retired | 3,000 - 3,999 | Condominium (excluding EC) | 0 | Smartphone, Tablet,  Laptop | Yes |
| EP04 | 69 | Female | Chinese | A-Level/ Diploma | Employed part-time | 1,000 -1,999 | Condominium (excluding EC) | 0 | Smartphone, Tablet,  Laptop | Yes |
| EP05 | 61 | Female | Chinese | Postgraduate | Retired | < 1,000 | Terrace House | 0 | Smartphone, Tablet,  Laptop | Yes |
| EP06 | 72 | Male | Chinese | Degree | Employed part-time | 7,000 - 7,999 | Condominium (excluding EC) | 1 | Smartphone, Tablet,  Laptop | Yes |
| EP07 | 68 | Female | Chinese | O-Level | Homemaker | 4,000 - 4,999 | Semi-Detached house | 1 | Smartphone,  Laptop | Yes |
| EP08 | 73 | Male | Chinese | A-Level/ Diploma | Retired | 4,000 - 4,999 | Semi-Detached house | 2 | Smartphone,  Laptop | Yes |
| EP09 | 68 | Female | Chinese | Degree | Retired | 0 | Condominium (excluding EC) | 2 | Smartphone,  Laptop | Yes |
| EP10 | 74 | Male | Chinese | A-Level/ Diploma | Retired | 0 | Condominium (excluding EC) | 3 | Smartphone,  Laptop | Yes |
| EP11 | 65 | Female | Chinese | Primary 6 or below | Employed part-time | < 1,000 | Rental block | 0 | Smartphone | Yes |
| EP12 | 71 | Female | Chinese | Secondary School | Retired | 0 | 4-room HDB flat | 2 | Smartphone, Tablet,  Laptop | Yes |
| EP13 | 55 | Female | Chinese | Degree | Employed part-time | < 1,000 | 4-room HDB flat | 0 | Smartphone,  Laptop | No |
| EP14 | 55 | Male | Chinese | A-Level/ Diploma | Employed full-time | 2,000 - 2,999 | 4-room HDB flat | 2 | Smartphone | No |
| EP15 | 62 | Male | Malay | N-Level/ITE | Employed full-time | 2,000 - 2,999 | 5-room HDB flat | 0 | Smartphone,  Laptop | Yes |
