# Supplementary material for: Health-Seeking Behavior and Its Associated Technology Use: Interview Study Among Community-Dwelling Older Adults
Source: JMIR Aging. 2023 May 4;6:e43709. doi: 10.2196/43709 (PMC10196894; doi:10.2196/43709)
Supplement: Multimedia Appendix 3 [file aging_v6i1e43709_app3.docx]

Multimedia Appendix 3. Sample responses regarding the preference and evaluation of health education

| factors | Sample responses |
| --- | --- |
| The qualification of the instructor | Personally, I prefer the health talks and tips given by the hospitals…Because the doctors are all on the panel, they could share among themselves and give you a good answer to any question you ask.  [EP05] |
| The language used by the instructor | The doctors try to reduce their professional terms, they try to use layman terms. I give them the credit that they do try, the terms are not so easy on them, but they do try to say in the way that we lay persons can understand, and I appreciate it.  [EP05] |
| The usefulness and relevance of the content | When a specialist is talking to you, they talk about very specialized subjects. For example, when they talk about kidney stones, they talk about how to manage kidney stones and how to manage [them], etc. They can pinpoint the problems…  [EP05]  They [the instructors] give me a lot of knowledge I need to know, they let me understand more in-depth what to eat and what not to eat, to exercise, talk to seniors, how to relate myself to the elderly like me or my parents, to encourage myself to move on.  [EP13]  I think the TCM [Traditional Chinese Medicine] class is useful, at least it gave me some understanding about how TCM can improve our health.  [EP14] |
| Cost | Normally I go down to Malaysia for courses as they are cheaper, e.g., how to take care of our back pain, what kind of exercise to do.  [EP15] |
| Personal interest | I more or less know the information given because I actually read quite a lot on health matters on the internet, I take health in my own hands. And I used to work in the health industry so I’m quite familiar, I can understand a bit more than what they are saying. That's where my interest lies, so if you read and you understand, it's easier for you to manage yourself, if you read and you don't understand, in the end, you will lose your motivation.  [EP09] |
