# Supplementary material for: Health-Seeking Behavior and Its Associated Technology Use: Interview Study Among Community-Dwelling Older Adults
Source: JMIR Aging. 2023 May 4;6:e43709. doi: 10.2196/43709 (PMC10196894; doi:10.2196/43709)
Supplement: Multimedia Appendix 4 [file aging_v6i1e43709_app4.docx]

Multimedia Appendix 4. Sample responses regarding evaluating the reliability of information sources

| methods of evaluating health information | Sample responses |
| --- | --- |
| Relying solely on official sources | I will go to HealthHub by the government.  [EP02] |
| Seeking verification from medical professionals | …I think it's still probably wise to consult a medical person. In my case, I’m fortunate to have my children who are medical[ly] trained. In the chat group, we do have doctors who are friends, so some of them will check in and advise us on whether it's true or not true, or what to do and what not to do.  [EP03] |
| Checking the credibility of information sources | Recently I have been following a guy on YouTube and I checked the credibility of the person who produced it, so now I’m exercising with him and subscribe[d] his YouTube channel for dumbbell exercises. So I actually checked his credibility and he is a renowned physiotherapist. I checked his background, and when I’m confident with his background, I become more confident in following the exercises that he teaches.  [EP05] |
| Searching for scientific evidence | I don't trust completely the websites. [It] depends on what website you see. I trust completely when I go to the library and borrow books. And the book is written in black and white, but I will also need to read the qualifications of the writer, whether the writer is a doctor or a practitioner. At the same time, sometimes when I see the doctor, I will also ask the doctor. I don't actually completely trust the authority. I used to argue with the polyclinic doctor, I got fatty liver and he said it's ok to have it and everyone has it, I said no I don't agree with you, the fatty liver is a sign of [a] lifestyle problem, you cannot say everyone has it so it's normal. When I questioned him, I told him I had done research about it. I will trust the specialists more because they do all the testing. I trust evidence, everything evidence-based.  [EP09] |
| Gathering information from individuals with similar experiences | Sometimes I chat with my friends about our health status and exchange health information.  [EP11]  I do my homework and get some information from the Internet, even though you cannot 100% trust the internet because sometimes there is so much conflicting information. I also get information from people who undergo all these kinds of diseases, I will ask them how do they get it, what do the doctors tell them, [and] what do the doctors advise them to do. So all this info is very handy as it's all from my friends.  [EP15] |
| Self-experiment | I didn't think about it until I talk to you now…If I cannot find any info about a medicine, I will still try it but will be very careful. The moment it gives me any negative result, I will stop it straight away. Actually, if I do it in this way, I become my own guinea pig, so I must be very careful.  [EP05]  I think it's based on experience, you must try it and then find oh it's true, if you don't try then don't bother. But there is no harm [in] trying. But I’m trying to be skeptical when people try to sell medicine, I try not [to] waste money buying some medicine and try, then later found it's not working.  [EP14] |
