# Supplementary material for: Health-Seeking Behavior and Its Associated Technology Use: Interview Study Among Community-Dwelling Older Adults
Source: JMIR Aging. 2023 May 4;6:e43709. doi: 10.2196/43709 (PMC10196894; doi:10.2196/43709)
Supplement: Multimedia Appendix 5 [file aging_v6i1e43709_app5.docx]

Multimedia Appendix 5. Sample responses regarding self-measurement

| Self-measurement | Reasons | Sample responses |
| --- | --- | --- |
| Constant self-measurement | **-** | I measure my blood pressure three times a day, this is the advice of my doctor from the polyclinic. I do that quite religiously and it's been very stable. I also monitor my weight and it's very stable as well.  [EP08] |
| Occasional self-measurement | **Awareness of bodily symptoms** | Only when symptoms come, I will look at it … If my BP is above 130, then I will monitor every day continuously for about a week, if it doesn't come down then I will go to the polyclinic.  [EP04] |
|  | **Awareness of daily activities** | When I eat a lot of sugar or go out to makan [eat], I will also check my blood sugar one hour after the consumption.  [EP15] |
| No self-measurement | **Lack of necessary equipment** | I used to have a blood pressure gauge, but somehow it's lost somewhere, and we are not able to take it out, so I haven't used it for quite some time. [EP14] |
|  | **Difficulty in obtaining accurate measurements** | Every time my own measurement is different from the one in the clinic.  [EP14] |
|  | **Perceived good health** | I don't use any tools to monitor my health at home. I don't have BP problem, and I have prediabetes, so I don't need to monitor. I believe the 10k steps walking can help me with that.  [EP09] |
|  | **Inability to schedule** | My doctor recommends me to measure my blood pressure every day at home, but a few years ago she gave me a chart to monitor my bp every day in the morning, in the afternoon and evening. I did the initial part but didn't follow up. My lifestyle is very good. Every morning I go for my very early walk at seven something, so I need to wake up early to eat my small breakfast. I need to leave the house by six something, so I never properly monitor my health.  [EP07] |
