# Supplementary material for: Health-Seeking Behavior and Its Associated Technology Use: Interview Study Among Community-Dwelling Older Adults
Source: JMIR Aging. 2023 May 4;6:e43709. doi: 10.2196/43709 (PMC10196894; doi:10.2196/43709)
Supplement: Multimedia Appendix 6 [file aging_v6i1e43709_app6.docx]

Multimedia Appendix 6. Sample responses regarding the impact of the COVID-19 pandemic on older people's perceived health

| Theme | Sample responses |
| --- | --- |
| The COVID-19 pandemic affects people’s routine exercise and dietary habits. | …I’m getting lazy these days because we've been living in the pandemic. After we stay home for long enough and we feel lazy to come out … I've become a good friend of ice cream and chocolate during the pandemic ... and my BMI is getting higher.  [EP05] |
| The COVID-19 pandemic causes more stress. | The COVID-19 down there is great stress… It affects a lot of people's job securities, especially my son who was running this rock climbing down there. All the activities were withheld, and the income will be curtailed…  [EP10] |
| The COVID-19 pandemic provides an opportunity to reflect on people’s life. | During COVID-19, I have more time to myself, more time to stabilize a lot of activities and stop a lot of things that are happening. I haven't done things like that. I’m able to take control of my thoughts and reflect [on] what I’m thinking, and to know what I want in life, what to do for the rest of my life. I don't have children, I’m married, and my husband is a family man that he goes to work. I’m at home for these two years during Covid-19, I spend more time accepting things as it is and to slow down things and to come to terms of myself in my mindset, also to cool down, calm down and know that life still has to carry on and need to mask up when you go out. Everything is normal to me and I feel that I’m able to take it calmly because I don't want to expose too much to the news of Covid-19 so much to the extent that I got phobia. So overall, I can say that I have more time to spend on myself, to do myself more these years, I've been running around doing work, doing chores. I feel that I have been, in other words, that I feel better, I feel my mind is more stabilized, and I’m able to cook things better than before. There are more than 2 years to rest, and my mental state is where I know what I want and [I am] very stable and I feel I have [a] healthy mindset. Rather than before Covid-19, I have to do things, keep moving on and moving on, to meet family members, to go for lunch with sister, to go to in-law’s house, going out without Covid. It made me be still and know who's my God in my life, and who is taking control of my life, know the government more, what they are doing for us citizens of Singapore.  [EP13] |
