# Supplementary material for: Health-Seeking Behavior and Its Associated Technology Use: Interview Study Among Community-Dwelling Older Adults
Source: JMIR Aging. 2023 May 4;6:e43709. doi: 10.2196/43709 (PMC10196894; doi:10.2196/43709)
Supplement: Multimedia Appendix 7 [file aging_v6i1e43709_app7.docx]

Multimedia Appendix 7. Sample responses regarding health service utilization

| responses to symptoms | Sample responses |
| --- | --- |
| Self-treatment | When I’m not feeling well, I just have a rest…I use my own "traditional" style, I drink more water when I’m getting flu or sore throat…I’ve practiced this traditional style for more than 30 years…Up to now, I’m still diagnosed free from my diabetes, high blood cholesterol.  [EP15] |
| Seeking prompt professional medical attention | See a doctor, [I] don’t buy medication from pharmacies.  [EP01] |
| Undergoing a logical process from self-treatment to professional medical attention, depending on the severity of symptoms | If it's more of tiredness and so on, then I will probably find time to have more rest, take a nap or go to bed early on in [the] afternoon, take a nap. If I’m still not feeling good like maybe having a headache or what, I may look to taking a Panadol or paracetamol. If [it is] a more serious problem, like recently a couple of months back I had self-tested positive on COVID, I will consult a doctor to find out what is the next step I have to do.  [EP03]  If for some reason I feel not so well, I'll give myself a break, meaning I will stay at home, just have a rest, be more conscious of the thing that I eat, means I will drink more water, eat more fruits. Because of so many years since my exercise regimen, I hardly see the doctor due to some health issue, maybe for those small issues like [an] ear infection. In fact, I try to avoid taking medication, [and] that's why I try to keep my lifestyle healthy. For bigger issues or the symptoms are not improving after a few days, I will go to see a family doctor who is a general practitioner.  [EP07] |
