# Supplementary material for: Health-Seeking Behavior and Its Associated Technology Use: Interview Study Among Community-Dwelling Older Adults
Source: JMIR Aging. 2023 May 4;6:e43709. doi: 10.2196/43709 (PMC10196894; doi:10.2196/43709)
Supplement: Multimedia Appendix 8 [file aging_v6i1e43709_app8.docx]

Multimedia Appendix 8. Sample responses regarding the choice of health technologies

| Theme | Sample responses |
| --- | --- |
| Perceived usefulness | The technology has to meet my need. [Healthy] 365 and HealthHub are just suitable for me, so I didn't go and look for other health apps. Some hospital also has an app, but whatever information in this app can be retrieved from HealthHub, so I don't use that app.  [EP04] |
| The comprehensiveness of features | For me, it has to be something that's inconvenient, it doesn't [I don’t] have to wear another device, for example, all [features] incorporated into one, like the basic thing: the steps count and somehow sleep pattern…  [EP03] |
| Perceived ease of use | I will just try, if it's not good and I don't know how to use, then I will delete.  [EP05] |
| Performance and quality | We started with the Relive [Relive: Run, Ride, Hike & more] app, but then I've noticed that sometimes we actually lost the satellite signal, and I complained to [the app developer] and they admit that they know they have such a problem. And then I use the Google Fit and Samsung Health, and usually the signals are very good.  [EP06] |
| Recommendation by the social network | I follow what the health and wellness course taught me, HealthHub is the first one, Healthy 365 is the second one. There are a few other apps they asked me to download, but I think HealthHub is the best.  [EP13] |
| Cost | In terms of the cost, I will check if it's free or paid.  [EP01]  First, I must say I’m a stingy woman, so if it's payable, I will think twice.  [EP05] |
| Rewards | To be frank, I don't mind using as many as possible, because they provide rewards.  [EP14] |
| Reliability | For reliability, I will check where it's produced/manufactured.  [EP01] |
